# Supplementary material for: Establishment and Molecular Characterization of Two Patient-Derived Pancreatic Ductal Adenocarcinoma Cell Lines as Preclinical Models for Treatment Response
Source: Cells. 2023 Feb 11;12(4):587. doi: 10.3390/cells12040587 (PMC9954561; doi:10.3390/cells12040587)
Supplement: Supplementary file 1 [file cells-12-00587-s001.zip › cells-2106007-supplementary.pdf]

Supplementary Figure S1

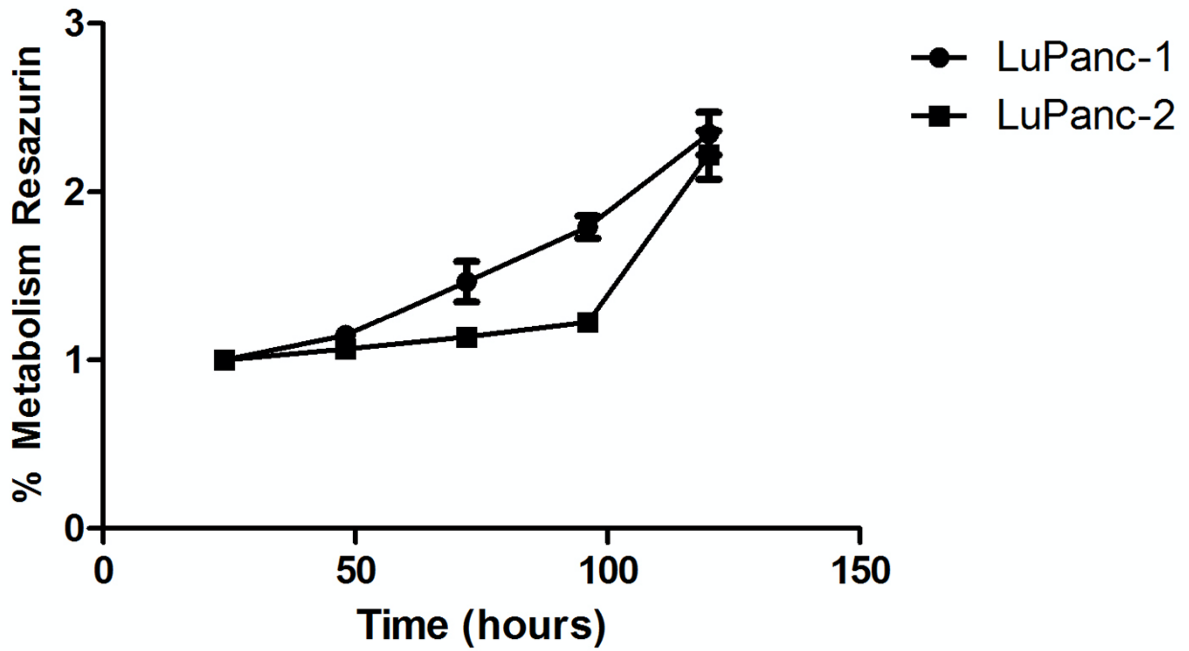

Figure S1. Cellular growth of LuPanc-1 and LuPanc-2 as measured by Resazurin reduction assay. **M**etabolic activity of LuPanc-1 and LuPanc-2 cells was measured for a total of 120 h. A doubling of metabolic activity was seen for LuPanc-1 cells after 74 h and for LuPanc-2 cells after 79 h.

Supplementary Figure S2

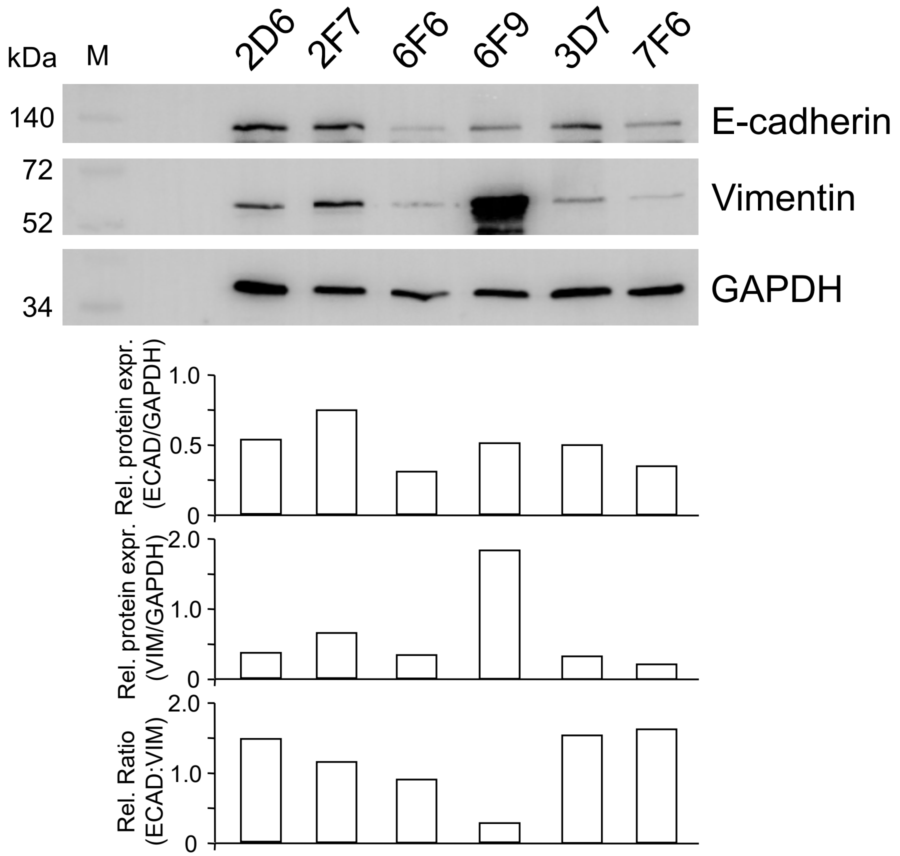

Figure S2. Immunoblot analysis of E-cadherin and vimentin in single cell-derived clonal cultures of LuPanc-1 cells. Proteinaceous extracts from the indicated LuPanc-1 clones were subjected to immunoblotting for E-cadherin, vimentin, and GAPDH to control for equal loading. The graphs underneath the blots indicate the results from a densitometry-based quantification of signal intensities. Data shown are representative of two experiments. Rel., relative; expr., expression. M, molecular weight marker.

Supplementary Figure S3: Enlargement of the oncoplot in Figure 3B (TP53, NUP153, PARN, genes A-C)

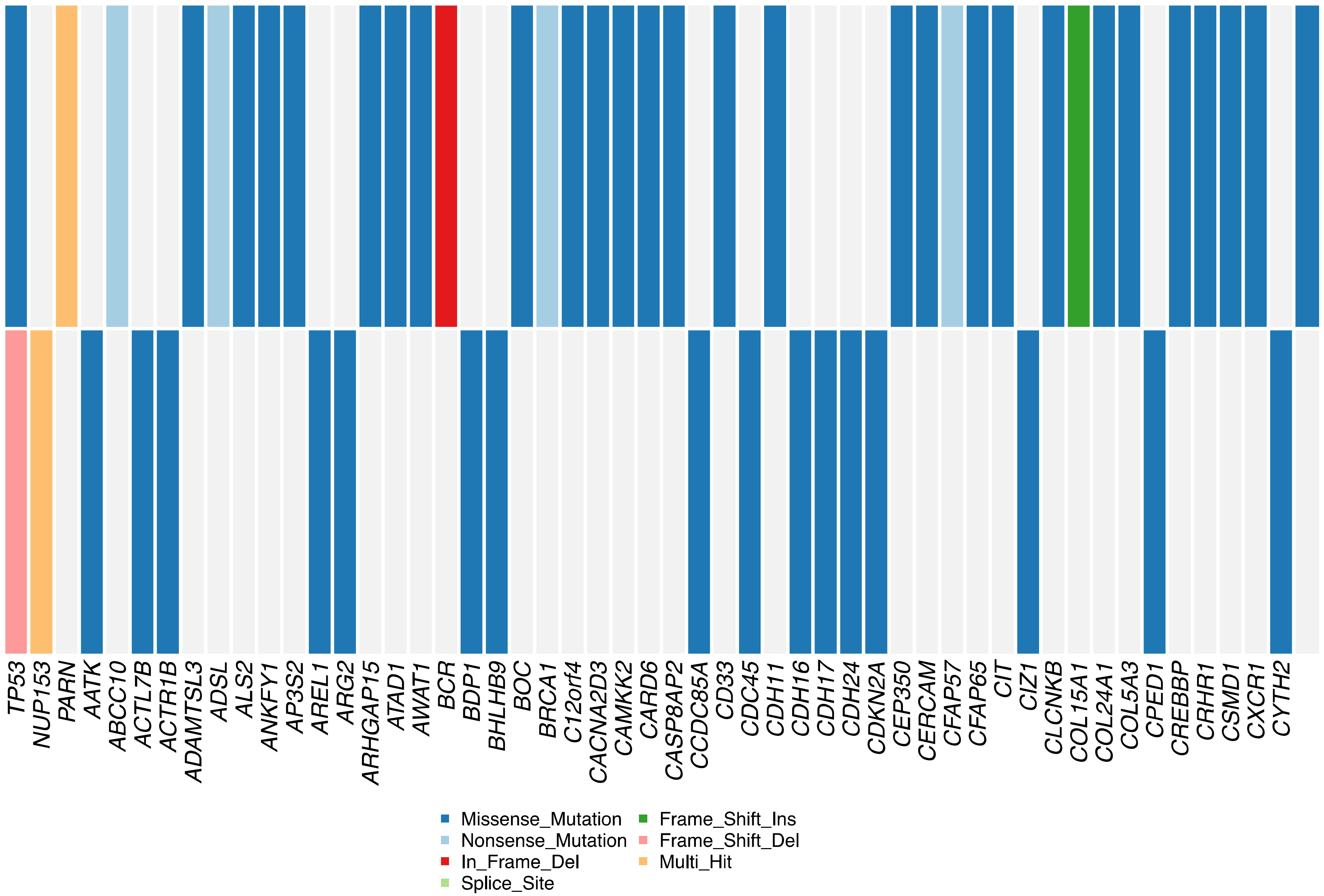

Supplementary Figure S3: Enlargement of the oncoplot in Figure 3B (Genes D-L)

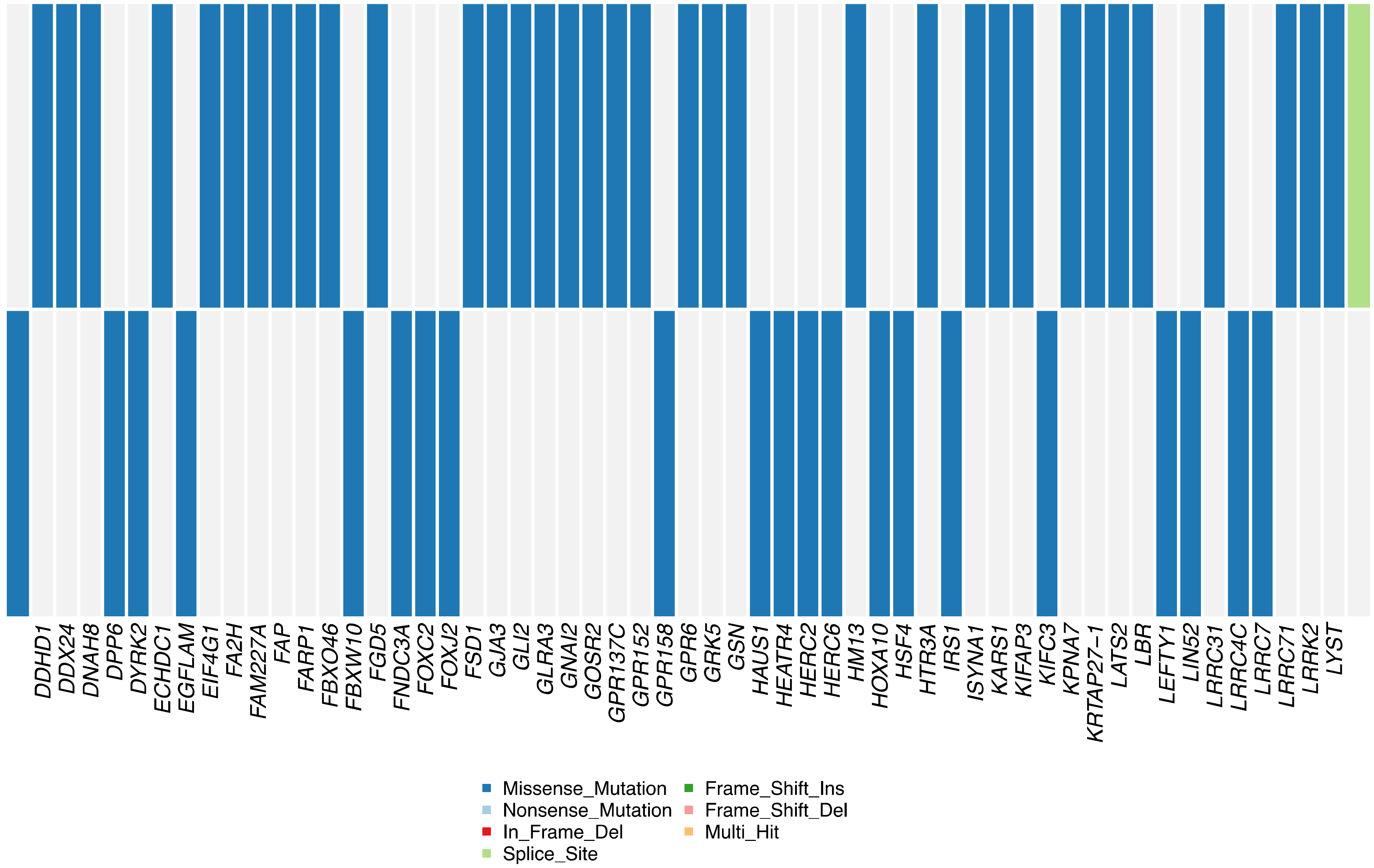

Supplementary Figure S3: Enlargement of the oncoplot in Figure 3B (Genes M-R)

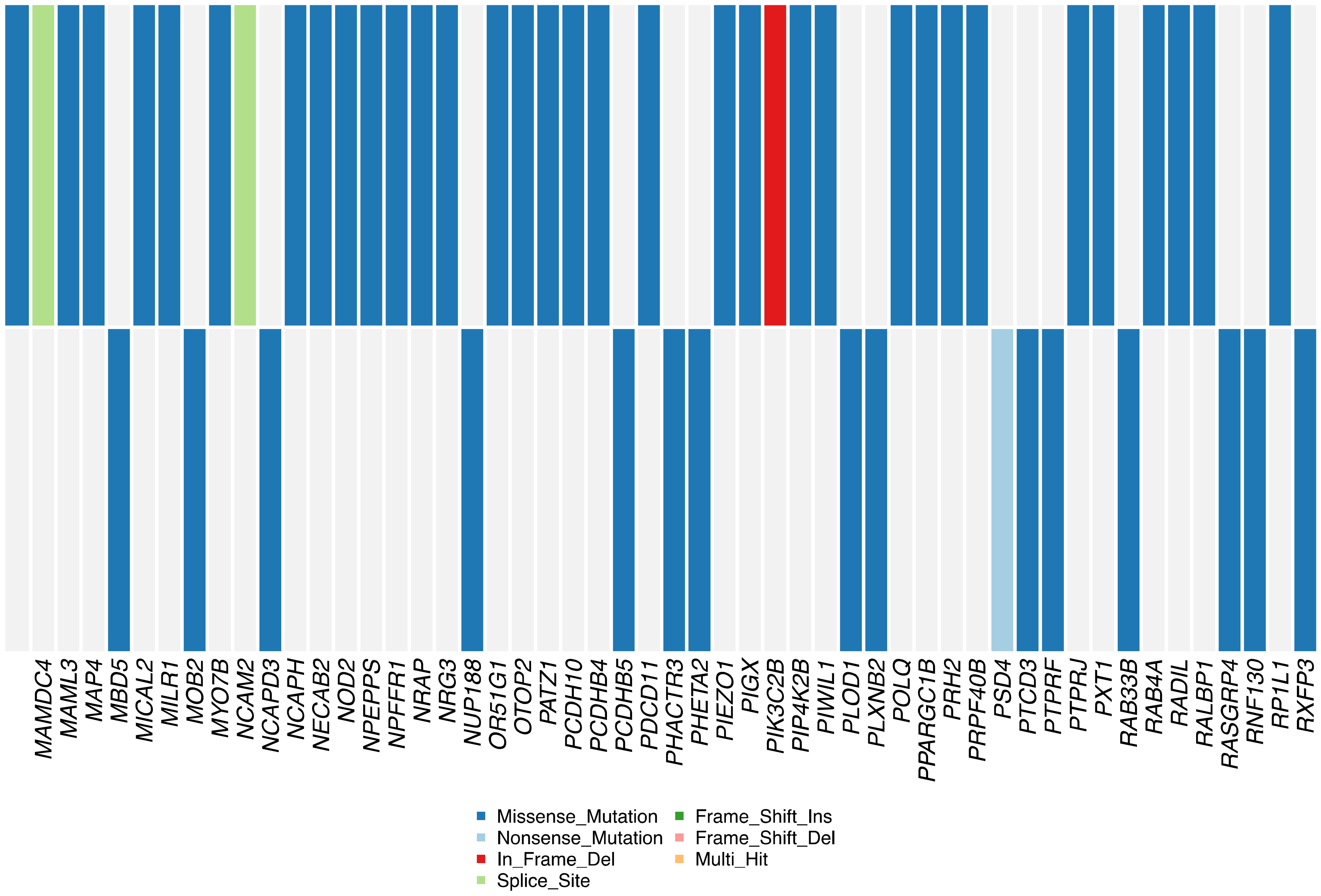

Supplementary Figure S3: Enlargement of the oncoplot in Figure 3B (Genes S-Z)

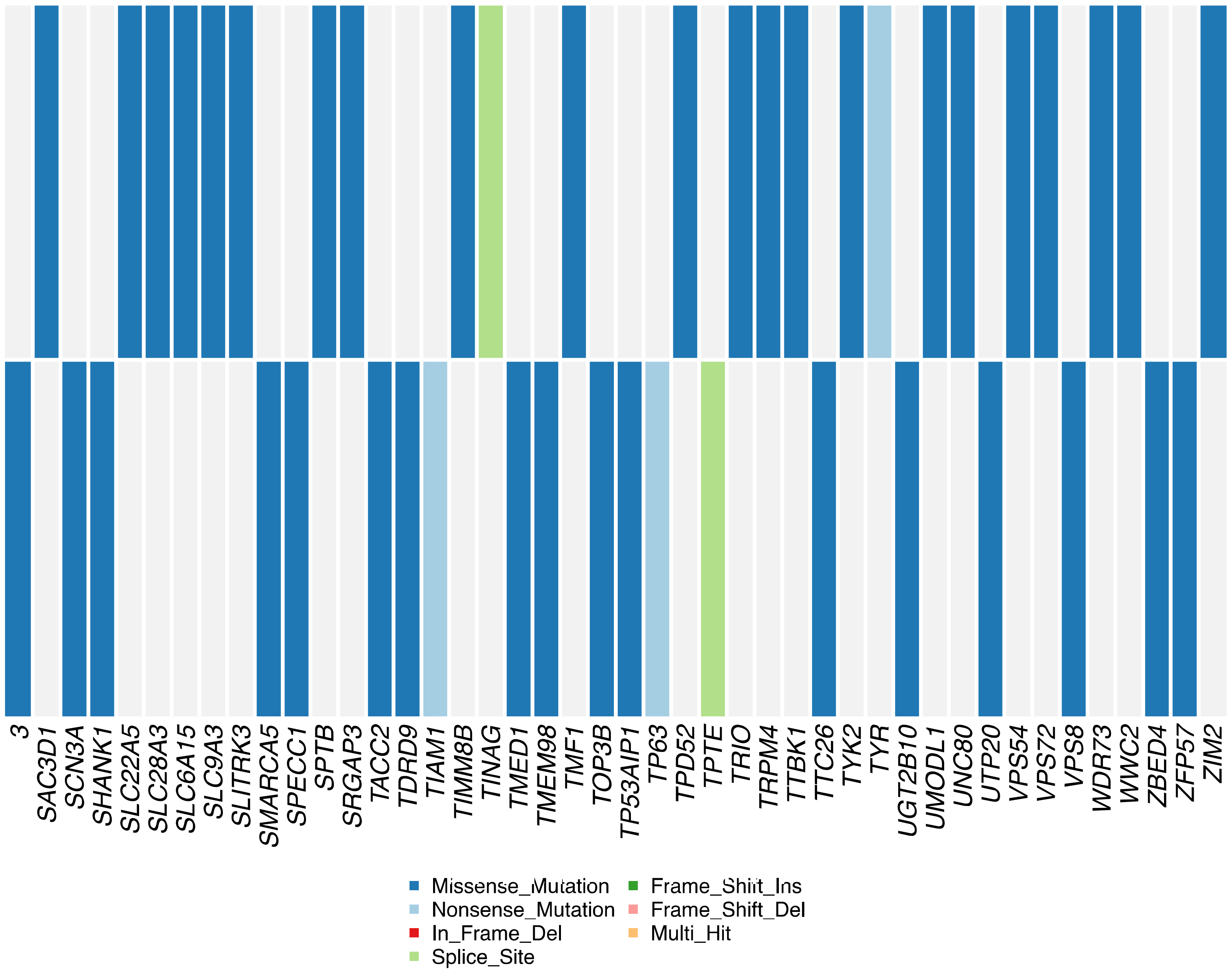

Supplementary Figure S4

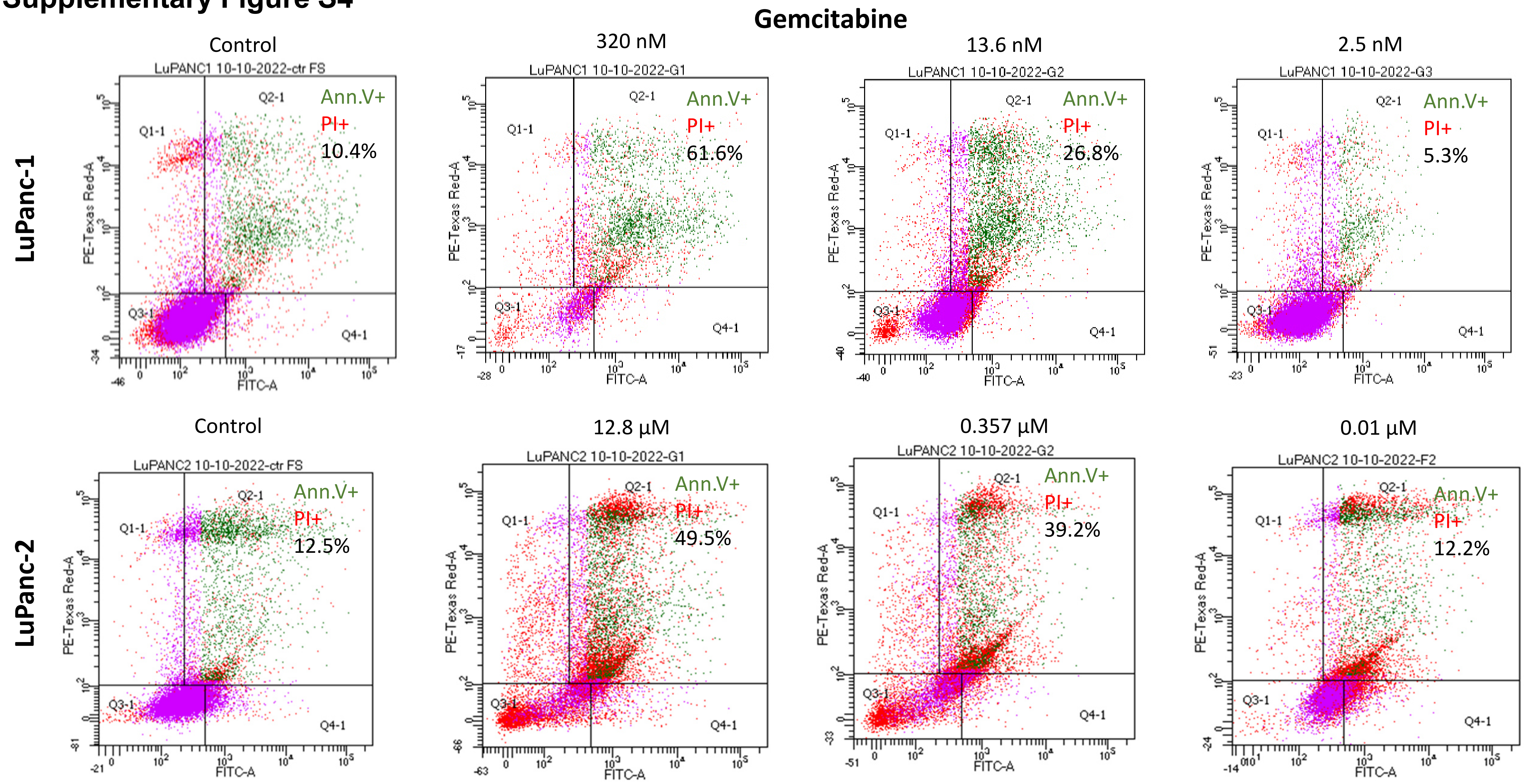

Figure S3. Apoptosis induction in LuPanc-1 (top row) and LuPanc-2 (bottom row) cells under gemcitabine treatment as measured by FACS analysis. The percentages of LuPanc-1 and LuPanc-2 cells positive for Annexin V (green) and PI (red) are selected in the upper right quarter. The respective drug concentration is given above each diagram.
